# Diseases of the Digestive Organs

**Published:** 1906-05-19

**Authors:** 


*(To be continued.)*

---

### DISEASES OF THE DIGESTIVE ORGANS.

**Gastric Dyspepsia.**—A classification of the functional disturbances of the stomach has recently been proposed by Hutchison,[1] who points out that much of the vagueness and confusion which surrounds the subject of dyspepsia is due to the fact that physicians have been content with describing its sub-groups by symptoms instead of by pathological processes. No doubt, in the evolution of our knowledge this has been the usual sequence of events, and very possibly the time has now arrived for scheduling our experiences of stomach disorders on more scientific lines. In place, then, of "acid dyspepsia," "atonic dyspepsia," "flatulent dyspepsia," and so on, Hutchison would have us divide the diseases of the stomach in which no gross lesion is present, according to the disturbance of function which is present. The stomach functions are secretory, motor, and sensory. Any of these may theoretically be disturbed in the direction of either diminution or augmentation, but practically there is probably no such condition as diminution of the sensibility of the stomach, as normally the stomach performs its work without producing any sensations in consciousness. Thus, we have five conditions to consider: (1) Increased secretion = hyperchlorhydria and continuous hypersecretion; (2) diminished secretion = hypochlorhydria and achylia gastrica; (3) increased motility = pyloric spasm and tormina ventriculi; (4) diminished motility = motor insufficiency leading to dilatation; (5) increased sensibility = gastralgia or hyperæsthesia of the stomach. Hutchison goes on to point out that these various groups are usually combined, decrease in motor power and secretory power, producing atonic or nervous dyspepsia with flatulence, increase of sensibility and secretion producing hypertonic dyspepsia, of which the chief symptom is pain. Hutchison, in common with all other modern authorities, rightly lays stress on the importance of a thorough examination of the stomach-contents by means of the stomach-tube. By this means the motor and secretory power of the stomach can be determined. He remarks that he has not found that patients as a rule object to the procedure, but the conditions under which the ordinary general practitioner finds himself are certainly different; and it may be doubted whether the public is at present prepared for this valuable diagnostic measure to the extent to which some specialists would have us believe. In dealing with the relationship between disturbance of function and symptoms Hutchison points out that without hyperæsthesia increased secretion probably produces no symptoms, and that the chief cause of serious dyspeptic symptoms is impairment of motor power. Decreased motility leads to flatulence, weight, discomfort, but not to actual pain; increased motility to "spasm" mainly in the pyloric region. Hutchison does not consider flatulence as due to microbic fermentation, and in this is in agreement with the eminent French writer, Soupault,[2] who says he considers stomach gases mainly arise from air-swallowing, partly from regurgitation through the pylorus, only slightly from fermentation, and not at all from exhalations from the blood-vessels in the stomach walls. As regards the hyperæsthetic condition of the mucous membrane, which Hutchison thinks the cause of pain in connection with hypersecretion of HCl, it is not clear whether his views correspond with those of Soupault. The latter is inclined to attribute the pain mainly to pyloric spasm, which can be relieved by gastrojejunostomy, though the causation of the spasm is probably hyperæsthesia of the mucosa. Hutchison insists strongly on the nervous disturbances which underlie functional derangements of the stomach, and on the whole discredits the view that errors in diet are a true cause of dyspepsia. In correspondence with this he relies in treatment on such general measures as rest in bed, regulated exercise, massage, etc., for relief of the symptoms rather than elaborately constructed dietaries.

[1] Brit. Med. Jour., Nov. 25, 1905, p. 1,381. [2] Brit. Med. Jour., Jan. 6, 1906, p. 23.

